# 583. *SARS-CoV-2* Spike Protein S1/S2 Antibodies after Vaccination with Sinopharm in Peruvian Physicians

**DOI:** 10.1093/ofid/ofab466.781

**Published:** 2021-12-04

**Authors:** Maria Lopera, Romina Vera, Carlos Tapia, Carlos Cabrera, Jose Gonzales-Zamora, jorge Alave

**Affiliations:** 1 Centro Nacional Salud Renal - Essalud, Lima, Lima, Peru; 2 Hospital Nacional Carlos Alberto Seguin Escobedo - Essalud, Arequipa, Arequipa, Peru; 3 University of Miami, Miller School of Medicine, Miami, Florida; 4 Clinica Good Hope, Lima, Lima, Peru

## Abstract

**Background:**

Peru started its national vaccination campaign in February 2021 using Sinopharm vaccine, targeting healthcare personnel on its initial phase. Although the immunogenicity of this vaccine was tested in clinical trials, there are no studies that evaluated the humoral response post vaccination in Peru.

**Methods:**

We conducted a cross sectional study, which objective was to evaluate the humoral immunogenicity triggered by the Sinopharm vaccine in Peruvian physicians. We collected demographic and epidemiologic data via an electronic. The SARS-CoV-2 spike protein S1/S2 antibodies were measured by chemiluminescense (Liaison®). A positive test was defined as >15 U/ml, which has correlation of 95% with neutralizing antibodies measured by plaque reduction neutralizing test.

**Results:**

92 participants were enrolled in the study. The epidemiologic characteristics are described in table 1. The mean level of antibodies measured at least 2 weeks from the second vaccine dose was 67.5 ± 70.5 U/ml. 85.7% of the study cohort had positive S1/S2 antibodies. In the univariate analysis, an imperfect negative correlation was found between the level of antibodies and participants’ age (r= -0.24; regression F test 5.25; p = 0.0242). A weak negative correlation was observed between the antibody titer and the time elapsed from the second vaccine dose and the day of antibody measurement (r= -0.17). A higher antibody level post vaccine was found in individuals who worked in COVID units (105.5 U / mL vs 58.2 U / mL; p = 0.0125), and in participants with history of COVID (216.5 U / mL vs 81.2 U / mL; p = < 0.0000). Hypertension was associated with lower antibody titers (36.9 U / mL vs. 74.6; p = 0.0464). In the multivariate analysis, working in COVID units, having previous COVID infection and shorter time from second vaccine dose and day of antibody measurement were associated with higher antibody levels post vaccine (table 2).

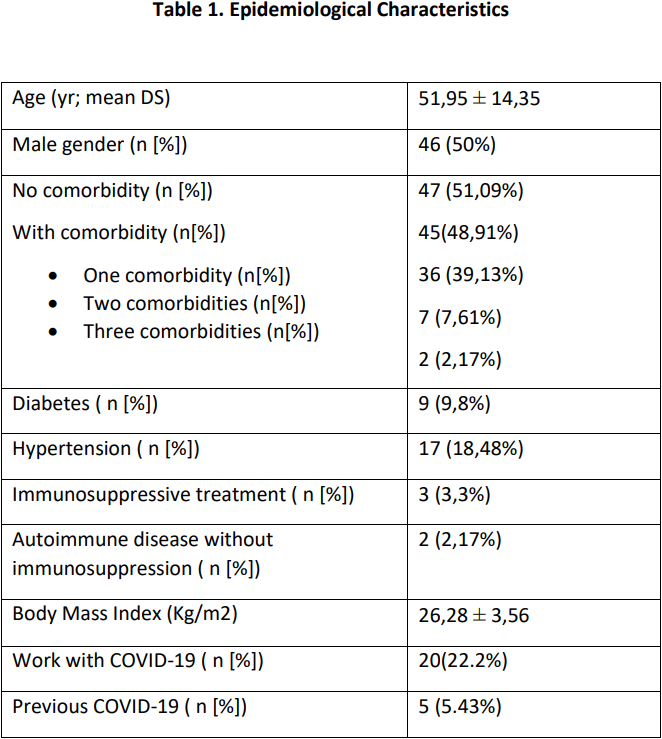

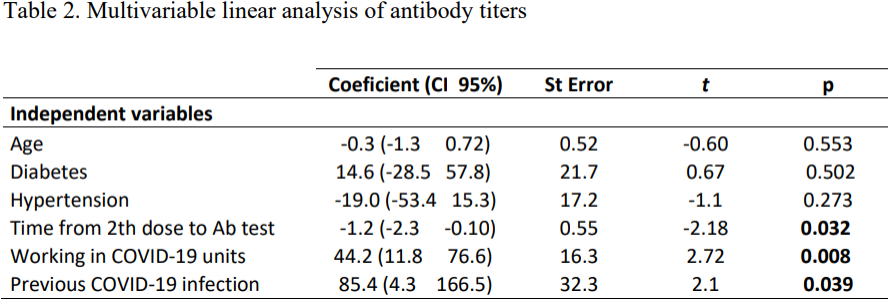

**Conclusion:**

Our study showed that the time elapsed from the second vaccine dose and the day of antibody measurement, having previous COVID-19 infection and working in COVID -19 units may help to predict higher antibody titers post vaccine. Larger studies to evaluate the humoral response post Sinopharm vaccine and its clinical implications are still needed in Peru.

**Disclosures:**

**All Authors**: No reported disclosures

